# Avian Introgression Patterns are Consistent With Haldane’s Rule

**DOI:** 10.1093/jhered/esac005

**Published:** 2022-02-03

**Authors:** Jente Ottenburghs

**Affiliations:** Wildlife Ecology and Conservation, Wageningen University & Research, Droevendaalsesteeg 3a, 6708PB Wageningen, The Netherlands; Forest Ecology and Forest Management, Wageningen University & Research, Droevendaalsesteeg 3a, 6708PB Wageningen, The Netherlands

**Keywords:** cline theory, coalescent modeling, genetic incompatibilities, genomics, hybridization, sex-biased dispersal

## Abstract

According to Haldane’s Rule, the heterogametic sex will show the greatest fitness reduction in a hybrid cross. In birds, where sex is determined by a ZW system, female hybrids are expected to experience lower fitness compared to male hybrids. This pattern has indeed been observed in several bird groups, but it is unknown whether the generality of Haldane’s Rule also extends to the molecular level. First, given the lower fitness of female hybrids, we can expect maternally inherited loci (i.e., mitochondrial and W-linked loci) to show lower introgression rates than biparentally inherited loci (i.e., autosomal loci) in females. Second, the faster evolution of Z-linked loci compared to autosomal loci and the hemizygosity of the Z-chromosome in females might speed up the accumulation of incompatible alleles on this sex chromosome, resulting in lower introgression rates for Z-linked loci than for autosomal loci. I tested these expectations by conducting a literature review which focused on studies that directly quantified introgression rates for autosomal, sex-linked, and mitochondrial loci. Although most studies reported introgression rates in line with Haldane’s Rule, it remains important to validate these genetic patterns with estimates of hybrid fitness and supporting field observations to rule out alternative explanations. Genomic data provide exciting opportunities to obtain a more fine-grained picture of introgression rates across the genome, which can consequently be linked to ecological and behavioral observations, potentially leading to novel insights into the genetic mechanisms underpinning Haldane’s Rule.

Haldane (1922) observed that “When in the F1 offspring of two different animal races one sex is absent, rare, or sterile, that sex is always the heterozygous [i.e. heterogametic] sex.” This observation applies to many groups of animals, regardless of whether the male or the female is the heterogametic sex (Orr 1997; Schilthuizen et al. 2011). In birds, where the sex is determined by a ZW system, females are the heterogametic sex and hybrid females are thus expected to show greater fitness reductions compared to hybrid males. This expectation has been confirmed for birds in general (Price and Bouvier 2002) and for specific bird groups, such as ducks (Tubaro and Lijtmaer 2002), wildfowl (Arrieta et al. 2013), and pigeons and doves (Lijtmaer et al. 2003). Despite the generality of Haldane’s Rule, the underlying mechanisms are still a matter of debate (Delph and Demuth 2016).

An often invoked explanation for Haldane’s Rule relies on dominance theory, which is based on the Dobzhansky–Muller incompatibility model (Wu and Ting 2004; Presgraves 2010). This theory states that hybrid sterility and unviability arise because of interactions between 2 or more genetic loci that have developed incompatible alleles during an allopatric phase. If these alleles are recessive and located on the Z-chromosome, their effect is expected to be more pronounced in female birds. This expectation is based on the fact that females lack another Z-chromosome that could carry a dominant version of the incompatible allele, which would nullify the negative effect of the recessive allele. The same reasoning applies to organisms with XY-chromosomes, where males carry the hemizygous Y-chromosome.

Another explanation for Haldane’s Rule can be traced back to work on *Drosophila* where substitutions on the X-chromosome have a larger impact on hybrid sterility and unviability than autosomal substitutions (Coyne and Orr 1989). This “Large X-effect” might be due to faster evolution of X-linked genes compared to genes on the autosomes. Accelerated evolution on the X-chromosome has been reported in several *Drosophila* species and certain mammals, but the overall evidence is mixed (Meisel and Connallon 2013). In birds, several studies have reported faster evolution of genes on the Z-chromosome (Mank et al. 2007; Wang, Ekblom et al. 2014), which has mainly been attributed to strong genetic drift due to its lower effective population size compared to autosomes and the accelerated fixation of non-dominant beneficial alleles which are exposed to selection on the hemizygous sex chromosome (Mank et al. 2010; Wright et al. 2015; Hayes et al. 2020). In some bird species, the direct effects of selection have been invoked to explain faster sequence divergence on the Z-chromosome (Hogner et al. 2012; Lavretsky et al. 2015). Particularly, a study on *Luscinia* nightingales suggested that strong postcopulatory sexual selection on males (with ZZ-chromosomes) might lead to lower levels of genetic diversity on the Z-chromosome. The consequent lower effective population size of the Z-chromosome will then render it more susceptible to random processes, such as genetic drift (Janoušek et al. 2018). In addition, the Z-chromosome experiences less recombination compared to autosomes, which could contribute to the accumulation of incompatible alleles on this sex chromosome due to the divergence of non-recombining sections (Butlin 2005; Wright et al. 2016). It is thus likely that multiple independent evolutionary processes can explain the “Faster Z-effect” in birds.

The initial observations of Haldane (1922) and the possible explanations for these patterns (e.g., dominance theory, Faster Z-effect) lead to several expected patterns on a molecular level, specifically with regard to the introgression rates of different genetic loci. First, because female bird hybrids are generally less fit than male hybrids, we can expect that maternally inherited loci (i.e., mitochondrial DNA and W-linked loci) will show lower levels of introgression compared to biparentally inherited loci. However, it is important to keep in mind that reduced introgression of maternally inherited loci can also be due to other processes, such as the stronger effect of drift (because of the smaller effective population size of mtDNA and W-linked loci compared to nuclear loci), the lack of recombination, or sex-specific differences in behavior or physiology (Ballard and Whitlock 2004; Patten et al. 2015; Evans et al. 2021). Therefore, it is crucial to also provide estimates of hybrid fitness before attributing lower introgression rates of maternally inherited loci to reduced fitness of female hybrids.

Second, the faster evolution of Z-linked loci compared to autosomal loci might speed up the accumulation of incompatible alleles on this chromosome. Moreover, if genes involved in premating and postzygotic isolation become physically linked on the Z-chromosome, they can facilitate the evolution of reproductive isolation. This situation has been suggested for *Ficedula* flycatchers where genes for low hybrid fitness and female preference seem to be located on the Z-chromosome (Saetre et al. 2003; Backström et al. 2010). The potential pivotal role of the Z-chromosome in reproductive isolation might thus result in lower introgression rates of Z-linked loci compared to autosomal loci (Irwin 2018). To investigate if 1) introgression rates of maternally inherited loci are lower compared to biparentally inherited loci due to reduced fitness of female hybrids, and if 2) introgression patterns of Z-linked loci are consistent with dominance theory as an explanation for Haldane’s Rule, I conducted a literature review in which I focused on papers that quantified and compared introgression rates for autosomal, sex-linked, or mitochondrial loci.

## Ninety Percent of Avian Studies Adhere to Haldane’s Rule

**Figure 1.**
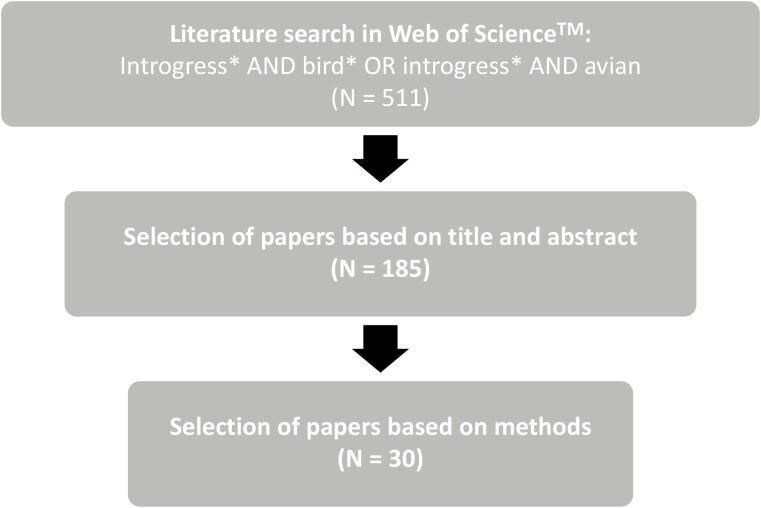
Overview of the search strategy and the number of studies in the literature review.

**Figure 2.**
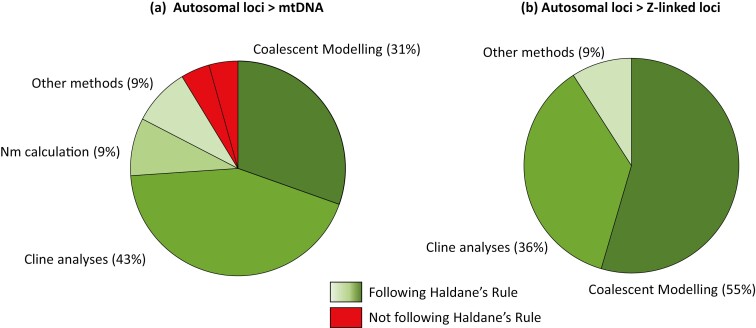
The literature review revealed that most studies reported introgression patterns in line with Haldane’s Rule, with (**a**) higher introgression of autosomal loci compared to mtDNA, and (**b**) higher introgression of autosomal loci compared to Z-linked loci. The studies used a variety of methods to infer patterns of introgression.

The literature search was done in 3 stages (Figure 1). First, I performed an extensive search on Web of Science™, using several broad keywords. The resulting set of 511 papers was subsequently scanned based on title and abstract to limit the number of studies with potentially useful information with regard to introgression rates and Haldane’s Rule. The remaining 185 studies were examined in more detail to extract data on introgression rates of autosomal, sex-linked, or mitochondrial loci. I applied a strict filter by only considering studies that provided direct estimates of introgression rates, using at least one of the following methods: coalescent modeling, cline analyses, or calculation of introgression from population genetic summary statistics. This search strategy uncovered 30 studies that directly compared introgression rates for autosomal, sex-linked, or mitochondrial loci (Table 1, Figure 2), using coalescent modeling (14 studies, 47%), geographic and genomic cline analyses of hybrid zones (10 studies, 33%), and calculating migration rates from genetic differentiation (3 studies, 10%). The majority of these studies documented introgression patterns in line with Haldane’s Rule (Table 1, 28 out of 30 studies, 93%). Specifically, 21 out of 23 studies (91%) reported introgression patterns of maternally inherited loci in line with Haldane’s Rule and 11 out of 11 studies (100%) reported lower introgression rates of Z-linked loci compared to autosomal loci (Figure 2).

**Table 1.** Overview of studies estimating introgression rates for different genomic classes

Species	Molecular markers	Introgression pattern	Haldane’s Rule	Hybrid fitness	Reference
*Nm* calculation					
* Phylloscopus collybita* * Phylloscopus brehmii*	mtDNA, microsatellites	Autosomal > mtDNA	Yes	No	Bensch et al. (2002)
* Aquila clanga* * Aquila pomarina*	mtDNA, AFLP	Autosomal > mtDNA	Yes	No	Helbig et al. (2005)
* Anas platyrhynchos* * Anas zonorhyncha*	Set of loci	mtDNA > autosomal	No	No	Kulikova et al. (2004)
Cline analyses					
* Manacus vitellinus* * Manacus candei*	mtDNA, nDNA	Autosomal > mtDNA	Yes	No	Brumfield et al. (2001)
* Pipilo maculatus* * Pipilo ocai*	mtDNA, AFLP	Autosomal > mtDNA	Yes	No	Kingston et al. (2012)
* Poecile atricapillus* * Poecile atricapillus*	Set of loci	Autosomal > mtDNA Autosomal > Z-linked	Yes	No	Sattler and Braun (2000)
	GBS	Autosomal > Z-linked	Yes	No	Taylor et al. (2014)
* Aphelocoma californica* (lineages)	mtDNA, microsatellites	Autosomal > mtDNA	Yes	No	Gowen et al. (2014)
* Jacana spinosa* * Jacana jacana*	Set of loci	Autosomal > mtDNA	Yes	No	Miller et al. (2014)
* Passer italiae* * Passer domesticus* * Passer hispaniolensis*	SNPs	Autosomal > mtDNA Autosomal > Z-linked	Yes	No	Hermansen et al. (2011)
* Ammodramus caudacutus* * Ammodramus nelsoni*	Microsatellites, set of loci	Autosomal > mtDNA Autosomal > Z-linked	Yes	Yes	Walsh et al. (2016)
* Pogoniulus chrysoconus extoni* * Pogoniulus pusillus pusillus*	mtDNA, microsatellites	Autosomal > mtDNA	Yes	No	Nwankwo et al. (2019)
* Baeolophus inornatus* * Baeolophus ridgwayi*	Set of loci	Autosomal > mtDNA	Yes	No	Cicero(2004)
Coalescent analyses					
* Aquila clanga* * Aquila pomarina*	Set of loci	Autosomal > Z-linked	Yes	No	Backström and Väli (2011)
* Passerina amoena* * Passerina cyanea*	Set of loci	Autosomal > mtDNA	Yes	No	Carling et al. (2010)
* Luscinia luscinia* * Luscinia megarhynchos*	Set of loci	Autosomal > Z-linked	Yes	Yes	Storchová et al. (2010)
* Tympanychus* species	Set of loci	Autosomal > Z-linked	Yes	No	Galla and Johnson (2015)
* Certhia americana* lineages	Set of loci	Autosomal > Z-linked	Yes	No	Manthey and Spellman (2014)
* Aegithalos bonvaloti* * Aegithalos fuliginosus*	Set of loci	mtDNA > autosomal mtDNA > Z-linked	No	No	Wang, Dai et al. (2014)
* Pheucticus melanocephalus* subspecies	Set of loci	Autosomal > mtDNA	Yes	No	van Els et al. (2014)
* Toxostoma lecontei* subspecies	Set of loci	Autosomal > Z-linked	Yes	No	Vázquez-Miranda et al. (2017)
* Aquila adalberti* * Aquila heliaca*	mtDNA, microsatellites	Autosomal > mtDNA	Yes	No	Martínez-Cruz and Godoy (2007)
* Anas crecca* * Anas carolinensis*	Set of loci	Autosomal > mtDNA	Yes	No	Peters et al. (2012)
* Sula nebouxii* * Sula variegata*	Set of loci	Autosomal > mtDNA Z-linked > mtDNA	Yes	No	Taylor et al. (2013)
* Zonotrichia capensis* subspecies	mtDNA, microsatellites	Autosomal > mtDNA	Yes	No	Cheviron and Brumfield (2009)
* Icterus galbula* * Icterus bullockii*	Set of loci	Autosomal > mtDNA	Yes	No	Jacobsen and Omland (2012)
* Passerina amoena* * Passerina cyanea*	Set of loci	Autosomal > mtDNA	Yes	No	Carling and Brumfield (2008)
Other methods					
* Gallus* species	Whole genome sequencing	Autosomal > Z-linked	Yes	No	Lawal et al. (2020)
* Hippolais icterina* * Hippolais polyglotta*	mtDNA, AFLP	Autosomal > mtDNA	Yes	No	Secondi et al. (2006)
* Poecile atricapillus* * Poecile carolinensis*	mtDNA, AFLP	Autosomal > mtDNA	Yes	No	Davidson et al. (2013)

Most studies applied coalescent modeling to quantify introgression rates for different genomic markers. This approach allows researchers to calculate introgression rates between populations or species within the framework of an Isolation-with-Migration (IM) model. These models use a Bayesian Markov chain Monte Carlo (MCMC) method to estimate population size, migration rate, and splitting time parameters (Hey and Nielsen 2004). By running IM models for different sets of genetic loci, it is possible to directly compare the resulting introgression rates for several genomic classes. For example, Carling and Brumfield (2008) used IM models to estimate introgression rates between lazuli bunting (*Passerina amoena*) and indigo bunting (*P. cyanea*) for autosomal, Z-linked, and mitochondrial loci. The results were in accordance with Haldane’s Rule, showing higher levels of introgression for autosomal loci compared to Z-linked  and mitochondrial ones. In general, most of the studies that applied coalescent modeling reported introgression patterns in line with Haldane’s Rule: 6 studies (out of 14, 43%) found higher introgression rates for autosomal loci compared to Z-linked loci, and 8 studies (57%) estimated higher introgression rates for autosomal loci than for mitochondrial loci.

Ten studies relied on cline analyses to estimate introgression rates for different genomic classes. Cline analyses quantify the changes in allele frequencies occurring along a geographic transect of a hybrid zone. According to cline theory, there is a straightforward relationship between selection against hybrids and cline width, namely that stronger selection against introgression results in a narrower cline (Slatkin 1973). Genomic loci with narrower clines might thus be more important in maintaining reproductive isolation between the hybridizing taxa than genomic loci with wider clines. With regard to Haldane’s Rule, it is expected that Z-linked and mitochondrial loci should exhibit narrower clines than autosomal loci. In total, 7 studies (out of 10, 70%) reported narrower clines for mitochondrial loci compared to autosomal loci, and 3 studies (30%) found that clines for Z-linked loci were narrower than clines for autosomal loci.

Another approach to estimate introgression rates relies on calculating *Nm* from genetic differentiation statistics, where *Nm* is the product of the effective population size (*N*) and the rate of migration (*m*) among populations (Slatkin 1985). In 2 out of 3 studies that applied this method, the introgression patterns followed the expectations from Haldane’s Rule. Bensch et al. (2002) found low mitochondrial introgression (*Nm* = 0.065) and high nuclear introgression (*Nm* = 4.9) between the common chiffchaff (*Phylloscopus collybita*) and the Iberian chiffchaff (*P. brehmii*). Similarly, introgression of nuclear loci (*Nm* = 1.1) was higher compared to mitochondrial loci (*Nm* = 0.11) for greater spotted eagle (*Aquila clanga*) and lesser spotted eagle (*A. pomarina*) (Helbig et al. 2005). However, *Nm* might be a less reliable proxy for introgression, because the calculation of this parameter depends on relative genetic differentiation, which can be influenced by other evolutionary processes.

Finally, 3 studies applied alternative methods to compare introgression rates between genomic classes. Using AFLP markers, Secondi et al. (2006) assigned individuals of icterine warbler (*Hippolais icterina*) and melodious warbler (*H. polyglotta*) to different parental and hybrid classes. This assignment exercise revealed introgression of autosomal loci, but indicated no exchange of mitochondrial loci. A similar approach in a hybrid zone between black-capped chickadee (*Poecile atricapillus*) and Carolina chickadee (*P. carolinensis*) revealed the same pattern: autosomal introgression, but no mitochondrial introgression (Davidson et al. 2013). A third study used data from whole genome sequencing to quantify introgression patterns between different *Gallus* species. Analyses based on D-statistics indicated lower introgression rates on the Z-chromosome compared to autosomal chromosomes (Lawal et al. 2020).

## Few Studies Directly Estimate Hybrid Fitness

The literature search revealed that most avian studies (90%) were in accordance with dominance theory as an explanation for Haldane’s Rule, confirming the generality of this pattern on the molecular level. However, just because a molecular pattern is in line with the expectations of Haldane’s Rule does not necessarily mean that reduced introgression of particular loci is due to the lower fitness of female hybrids. Other mechanisms, such as mate choice, local selection pressures, or sex-biased dispersal, can account for these patterns (Petit and Excoffier 2009). To confidently invoke Haldane’s Rule, one has to provide convincing evidence for reduced fitness of female hybrids (e.g., Neubauer et al. 2014). Across all studies that reported genetic patterns in accordance with Haldane’s Rule, only 2 studies explicitly referred to direct estimates of hybrid fitness (Table 1). Storchová et al. (2010) mentioned that captive crosses between thrush nightingale (*Luscinia luscinia*) and common nightingale (*L. megarhynchos*) yielded sterile females and fertile males (Stadie 1991). Similarly, a study on a hybrid zone between saltmarsh sparrow (*Ammodramus caudacutus*) and Nelson’s sparrow (*A. nelsoni*) indicated that hybrid females had lower survival rates than hybrid males (Walsh et al. 2016). Hence, most studies reported genetic patterns in line with Haldane’s Rule, but did not confirm the underlying mechanism with additional data on hybrid fitness.

The lack of hybrid fitness estimates is not surprising as this parameter can be extremely difficult and cumbersome to measure in wild populations (Linnen and Hoekstra 2009). Moreover, hybrid individuals might be rare (Taylor et al. 2012) or only occasionally observed in the field (Galla and Johnson 2015). Some exceptions include study systems where researchers can rely on long-term population data to connect introgression rates with fitness parameters (Grant and Grant 2016; Lamichhaney et al. 2020) or species that can be bred in captivity to quantify the fertility of male and female hybrids (Stadie 1991; Lijtmaer et al. 2003; Campagna et al. 2018). Given the difficulty of estimating hybrid fitness, some researchers might be tempted to turn to genomic data because methods to infer introgression and selection are constantly improving (Ottenburghs et al. 2017; Setter et al. 2020). However, solely relying on genomic data can potentially lead to wrong conclusions, which was nicely illustrated by a recent study on Darwin’s Finches. Genomic analyses of the medium ground finch (*Geospiza fortis*) and the cactus finch (*G. scandens*) pointed to reduced introgression on the Z-chromosome in comparison with autosomal loci. This pattern might be due to genetic incompatibilities on the Z-chromosome (which would support Haldane’s Rule), but detailed field observations showed that particular mating patterns—unrelated to female fertility—could explain the introgression rates (Lamichhaney et al. 2020). This example highlights the value of field observations and indicates the importance of combining different lines of evidence to determine whether Haldane’s Rule holds for a particular study system.

## Exceptions to Haldane’s Rule: Sex-Biased Dispersal and Ancient Introgression

Only 2 studies reported introgression patterns that were not in line with Haldane’s Rule. Kulikova et al. (2004) found that hybridization between mallard (*Anas platyrhynchos*) and spot-billed duck (*A. zonorhyncha*) resulted in higher introgression rates for mitochondrial loci compared to autosomal loci. This pattern can be explained by asymmetrical mating patterns and sex-biased dispersal between these duck species. Male spot-billed ducks tend to disperse more than the philopatric female spot-billed ducks. Hence, male spot-billed ducks are more likely to come into contact with female mallards. Consequently, most hybridization events involved a male spot-billed duck and a female mallard. When the resulting hybrids return to the breeding grounds of spot-billed ducks, they carry the mallard mtDNA with them, leading to asymmetrical introgression of mtDNA from mallards into spot-billed ducks. In contrast, nuclear introgression is mediated by both males and females, so nuclear loci are not expected to reflect dispersal differences between the sexes. In this case, introgression rates for nuclear loci were lower than introgression rates for mitochondrial ones, although still primarily from mallards into spot-billed ducks. This exception to Haldane’s Rule emphasizes the importance of understanding the life history and behavioral characteristics of the study system.

The second exception concerns high levels of mitochondrial introgression between black-browed tit (*Aegithalos bonvaloti*) and sooty tit (*A. fuliginosus*) compared to negligible exchange of autosomal and Z-linked loci (Wang, Dai et al. 2014). The authors attributed this pattern to ancient introgression of mtDNA, potentially in combination with a selective sweep or strong genetic drift. Ancient introgression events have been uncovered in several avian families (Fuchs et al. 2013; Ottenburghs et al. 2017; Zhang et al. 2021), sometimes even involving extinct lineages (Zhang et al. 2019; Ottenburghs 2020). In addition, mtDNA is known to easily cross species boundaries and quickly spread throughout a population (Funk and Omland 2003; Toews and Brelsford 2012). The fixation of a mitochondrial variant can often be explained by genetic drift as the effective population size of mtDNA is only one quarter of that of nuclear DNA (Ballard and Whitlock 2004). In some cases, the introgressed mtDNA might confer an adaptive advantage on the receiving species, speeding up the fixation process (Morales et al. 2017). Regardless of the underlying mechanism, ancient introgression of mtDNA can lead to introgression patterns that deviate from Haldane’s Rule.

## Haldane’s Rule in the Genomic Era

Despite the increasing availability of genomic data for birds, only one study used a genomic approach to directly estimate introgression rates (Lawal et al. 2020). The lack of genomic studies in this literature review can be explained by 1) my strict search strategy and 2) the focus of recent genomic studies on finding loci involved in reproductive isolation instead of estimating introgression rates. First, my literature search only included studies that directly quantified introgression rates, using approaches such as coalescent modeling and cline analyses. Most genomic studies indirectly inferred introgression by comparing levels of genetic divergence between different genomic regions. For example, Stryjewski and Sorenson (2017) suggested autosomal introgression in *Lonchura* munias based on a limited number of divergent autosomal regions, while mitochondrial introgression was deemed less likely based on the observation of distinct mitochondrial haplotypes. Although these patterns are consistent with Haldane’s Rule, they could also be explained by the higher mutation rates in mtDNA (Ballard and Whitlock 2004) or selective sweeps on autosomal loci (Hejase et al. 2020). In addition, methodological issues in assembling sex chromosomes from genomic data—which often have lower coverage—might result in biased estimates of genetic differentiation and consequent inferences of introgression rates (Okuno et al. 2021). Based on these theoretical and methodological uncertainties, I decided to omit studies that infer introgression patterns from genetic divergence, resulting in the removal of several genomic studies from the literature search.

A second explanation for the lack of genomic studies concerns the recent focus in speciation genomics on detecting barrier loci that might contribute to reproductive isolation (Ravinet et al. 2017; Campbell et al. 2018). The comparison of genomes between closely related species has revealed a heterogenous genomic landscape in which genetic differentiation is often distributed in particular genomic regions (Wolf and Ellegren 2017). These “genomic islands of differentiation” might contain loci involved in reproductive isolation, shielding them from the homogenizing effects of introgression (Turner et al. 2005; Ravinet et al. 2017). However, other evolutionary processes, such as linked selection, can also explain the formation of genomic islands (Battey 2019; Rettelbach et al. 2019). Consequently, recent research efforts have been mainly aimed at understanding  the evolutionary forces underlying this heterogenous genomic landscape (Stryjewski and Sorenson 2017; Bourgeois et al. 2020; Ottenburghs et al. 2020; Sendell-Price et al. 2021), rather than directly estimating introgression rates for particular genomic regions (but see Schield et al. 2021). In addition, most genomic studies that did quantify introgression rates between hybridizing species provided a genome-wide estimate instead of comparing genomic classes (Pulido-Santacruz et al. 2019; Ottenburghs et al. 2020; Sadanandan et al. 2020). Future genomic studies could focus more on relating introgression rates of certain genomic regions to particular fitness components, potentially providing more insights into the genetic mechanisms underlying Haldane’s Rule.

Finally, no study included W-linked loci in the analyses. The W-chromosome is notoriously difficult to sequence due to its haploid nature and high repeat content (Tomaszkiewicz et al. 2017). However, the development of new sequencing technologies for non-model organisms will allow researchers to explore the evolutionary history of this sex chromosome (Xu et al. 2020; Rogers et al. 2021) and its connection to Haldane’s Rule. For example, a recent study reported an excess of endogenous retroviruses (ERVs) on the W-chromosome, which could contribute to genetic incompatibilities between species due to mismatches in ERV-repressor mechanisms in hybrids (Peona et al. 2021). Quantifying introgression patterns of W-linked loci provides exciting avenues for future research.

These knowledge gaps—estimating local introgression rates across the genome and studying the W-chromosome—can be addressed as genomic resources and methods to quantify introgression are constantly improving (Bravo et al. 2021; Hibbins and Hahn 2021). A more fine-grained picture of introgression rates across the genome will lead to important insights into the evolutionary processes and genomic features that determine whether introgressed loci will be retained or removed from the population (Runemark et al. 2019; Moran et al. 2020). Linking these introgression patterns to fitness estimates and field observations might result in novel insights on the mechanistic basis of Haldane’s Rule.

## Data Availability

All the data associated with this paper can be found in Table 1.
